# Autoantibodies neutralizing type I IFNs in a fatal case of H5N1 avian influenza

**DOI:** 10.1084/jem.20251962

**Published:** 2025-12-05

**Authors:** Qian Zhang, Taylor S. Conrad, Marcela Moncada-Velez, Kaijun Jiang, Anastasija Cupic, Jonathan Eaton, Kimberley Hutchinson, Adrian Gervais, Ruyue Chen, Anne Puel, Paul Bastard, Aurelie Cobat, Theresa Sokol, Ryan A. Langlois, Lisa Miorin, Adolfo García-Sastre, John A. Vanchiere, Jean-Laurent Casanova

**Affiliations:** 1 St. Giles Laboratory of Human Genetics of Infectious Diseases, Rockefeller Branch, The Rockefeller University, New York, NY, USA; 2 Laboratory of Human Genetics of Infectious Diseases, Necker Branch, INSERM U1163, Necker Hospital for Sick Children, Paris, France; 3 Université Paris Cité, Imagine Institute, Paris, France; 4 Section of Pulmonary and Critical Care Medicine, Department of Medicine, LSU Health Shreveport, Shreveport, LA, USA; 5 Department of Emergency Medicine, LSU Health Shreveport, Shreveport, LA, USA; 6 Department of Microbiology, Icahn School of Medicine at Mount Sinai, New York, NY, USA; 7 Graduate School of Biomedical Sciences, Icahn School of Medicine at Mount Sinai, New York, NY, USA; 8 Department of Nephrology and Immunology, Children’s Hospital of Soochow University, Suzhou, China; 9 Pediatric Hematology-Immunology and Rheumatology Unit, Necker Hospital for Sick Children, Assistance Publique-Hôpitaux de Paris (AP-HP), Paris, France; 10 Louisiana Department of Health, Baton Rouge, LA, USA; 11 Department of Microbiology and Immunology, University of Minnesota, Minneapolis, MN, USA; 12 Global Health and Emerging Pathogens Institute, Icahn School of Medicine at Mount Sinai, New York, NY, USA; 13 Department of Medicine, Division of Infectious Diseases, Icahn School of Medicine at Mount Sinai, New York, NY, USA; 14 The Tisch Cancer Institute, Icahn School of Medicine at Mount Sinai, New York, NY, USA; 15 Department of Pathology, Molecular and Cell-Based Medicine, Icahn School of Medicine at Mount Sinai, New York, NY, USA; 16 The Icahn Genomics Center, Icahn School of Medicine at Mount Sinai, New York, NY, USA; 17 Section of Pediatric Infectious Diseases, Department of Pediatrics, LSU Health Shreveport, Shreveport, LA, USA; 18 Howard Hughes Medical Institute, New York, NY, USA

## Abstract

Avian influenza A virus (IAV) H5N1 is an emerging threat of human pandemic. We describe a 71-year-old man who died of H5N1 pneumonia in Louisiana and whose blood contained autoantibodies neutralizing type I IFNs (AAN-I-IFNs), including the 12 IFN-α subtypes (1–10 ng/ml) and IFN-ω (100 pg/ml). Causality between these AAN-I-IFN and lethal outcome of avian influenza in this patient is based on (1) our previous report that AA-I-IFN underlie about 5% of cases of critical pneumonia triggered by seasonal influenza viruses in three cohorts, (2) the rarity of this combination of AAN-I-FNs in individuals over 70 years old (<1%), and (3) the rarity of lethal avian influenza among infected individuals (<1%). AAN-I-IFNs underlie a growing number of severe viral diseases, from arboviral encephalitis to viral pneumonia, particularly in the elderly. This case suggests they can also underlie life-threatening avian H5N1 influenza. The presence of AAN-I-IFN may facilitate infection, replication, and adaptation of zoonotic IAVs to humans and, therefore, human-to-human transmission.

## Teaser

Zhang et al. report a fatal case of avian influenza H5N1 infection caused by autoantibodies neutralizing type I IFNs. These rogue autoantibodies are common, especially in elderly people. They are found to underlie a growing range of severe infections of emerging and circulating viruses, including seasonal influenza viruses and SARS-CoV-2.

## Graphical Abstract

**Figure:**
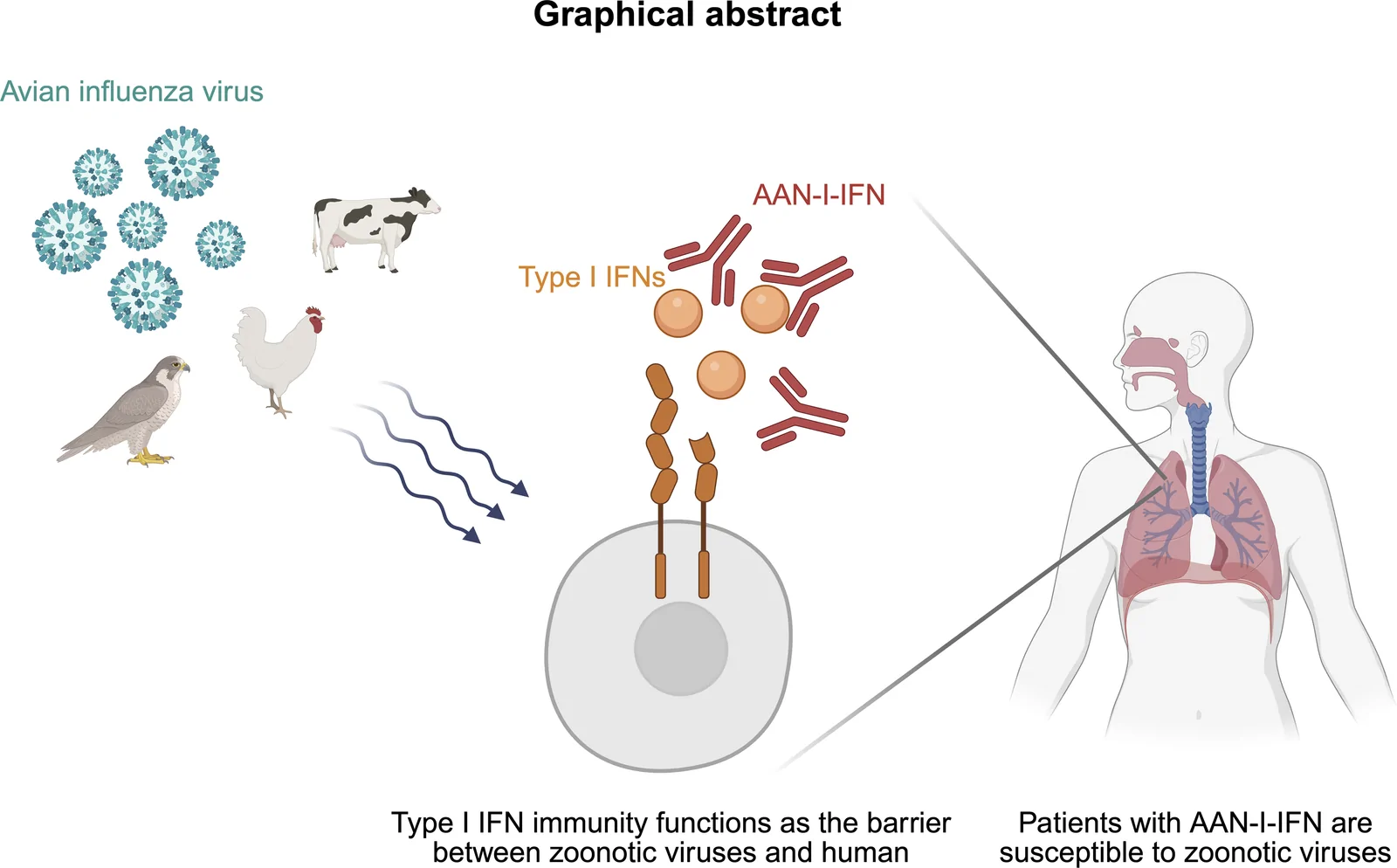

## Introduction

Influenza A viruses (IAVs) triggered the 1918 Spanish flu (H1N1), 1957 Asian flu (H2N2), 1968 Hong Kong flu (H3N2), 1977 Russian flu (H1N1), and 2009 swine flu (H1N1) epidemics and pandemics. Zoonotic IAVs, especially H5N1 and H7N9, are among the greatest current threats to public health due to their pandemic potential (WHO, 2025; CDC, 2025). H5N1 IAV causes lethal infections in poultry, leading to important economic losses, but can also infect various mammals, including humans, causing severe disease. Since 1997, 985 cases of H5N1 infection in humans have been recorded in 25 countries by the WHO, with a fatality rate of 48% (case-fatality ratio) (WHO, 2025). From 2013 to 2019, 1,500 human cases of H7N9 infection were reported, all in China, with a fatality rate of 40% (Chen et al., 2021). Data for estimating the true infection-fatality rate (IFR) of H5N1 and H7N9 IAV infections are scarce, but this rate is probably below WHO estimates (WHO, 2025) because the rate of seroconversion in exposed individuals is only 1–2%, and even if infected, many mild cases remain undiagnosed (Wang et al., 2012). Indeed, recent studies on human H5N1 infections showed that all cases were asymptomatic or mild (Garg et al., 2025; Shimizu et al., 2016). Similar findings were reported for humans infected with H7N9 (Chen et al., 2014). So far, no human-to-human transmission of either avian IAV has been documented. However, the recent introduction of H5N1 viruses into cows resulted in several H5N1 infections in dairy workers, probably through exposure to infectious material from infected cow’s milk (Garg et al., 2025). As with other infectious agents, there is immense interindividual variability in humans exposed to H5N1 or H7N9, ranging from silent infection to lethal disease, and the IFR is not precisely known (Casanova and Abel, 2022).

Protection against avian IAVs may be mediated by cross-reactive adaptive immunity to seasonal IAVs, or by intrinsic or innate immunity. Since 2015, we and others have reported that rare monogenic inborn errors of type I IFN immunity can underlie life-threatening seasonal IAV infections in otherwise healthy individuals (Ciancanelli et al., 2015; Zhang et al., 2022a; Hernandez et al., 2018; Lim et al., 2019). Moreover, variants of *MX1*, an IFN-stimulated gene (ISG) that is functional in humans but not birds, were identified in Chinese individuals who developed severe H7N9 infection (Chen et al., 2021). Since 2020, we have also shown that autoantibodies neutralizing type I IFNs (AAN-I-IFNs) can underlie 5% of severe seasonal influenza pneumonia (Zhang et al., 2022b), 10% of tick-borne encephalitis (Gervais et al., 2024b), 15% of hypoxemic COVID-19 pneumonia (Bastard et al., 2020, 2021a; Stertz and Hale, 2021), 20% of severe Middle East respiratory syndrome pneumonia cases (Alotaibi et al., 2023), 30% of severe adverse reactions to live-attenuated yellow fever virus vaccine (YFV-17D) (Bastard et al., 2021b), 40% of West Nile virus encephalitis cases (Gervais et al., 2023), and most cases of the rarer Usutu or Powassan virus encephalitis (Gervais et al., 2024a). Unlike most known inborn errors of type I IFN immunity, AAN-I-IFNs are common in the general population, with a prevalence of about 0.5% under the age of 70 years and about 5% over the age of 70 years (Bastard et al., 2021a). Samples from most patients (1:10 diluted plasma or serum) neutralize 0.1–10 ng/ml IFN-α and/or -ω, resulting in a very high odds ratio (OR: 100–500) for severe disease relative to controls with mild/asymptomatic infection (Bastard et al., 2024). We thus tested the hypothesis that AAN-I-IFN can underlie severe H5N1 infection.

## Results and discussion

On December 9, 2024, a 71-year-old man of European descent with a 1-week history of dyspnea, fever, and confusion presented at a rural hospital in Jennings, LA. On admission, the patient had acute hypoxic respiratory failure. Chest X ray revealed multifocal pneumonia involving the right upper and lower lobes. The patient was intubated and transferred to the ICU on day 1 of hospitalization (day 1). On day 2, IAV was detected in swabs (influenza A/Louisiana/12/2024 H5N1 virus, D1.1 genotype). Oseltamivir was initiated on day 2, and baloxavir was added on day 3. The patient kept domesticated chickens and ducks, which had died about 5–7 days earlier. He was transferred to the Academic Medical Center of Ochsner-LSU Health Shreveport in Shreveport, LA. On day 3, veno-venous extracorporeal membrane oxygenation was initiated, and therapeutic plasma exchange (TPE) was performed due to concerns about thrombotic microangiopathy and viremia. Continuous renal replacement therapy (CRRT) was performed from days 4 to 13 and days 20 to 28, and an ExThera Seraph 100 Microbind Affinity Blood Filter was used (days 4 to 8). The course of the infection was complicated by atrial fibrillation with rapid ventricular response and gastrointestinal bleeding. Tracheostomy was performed on day 14. On day 25, the patient developed profound hypoxia and hypercapnia, with anuria and a return of vasoplegic shock, which became refractory to treatment. On day 29, a transition to comfort care was decided. The patient died shortly thereafter, surrounded by family. During hospitalization, the patient was tested positive for cold agglutinin, anti-Kna (Knops), and anti-JKB antibodies. He had no clinical history of severe infectious diseases or autoimmunity and no recorded influenza or COVID-19 vaccination.

**Figure 1.**
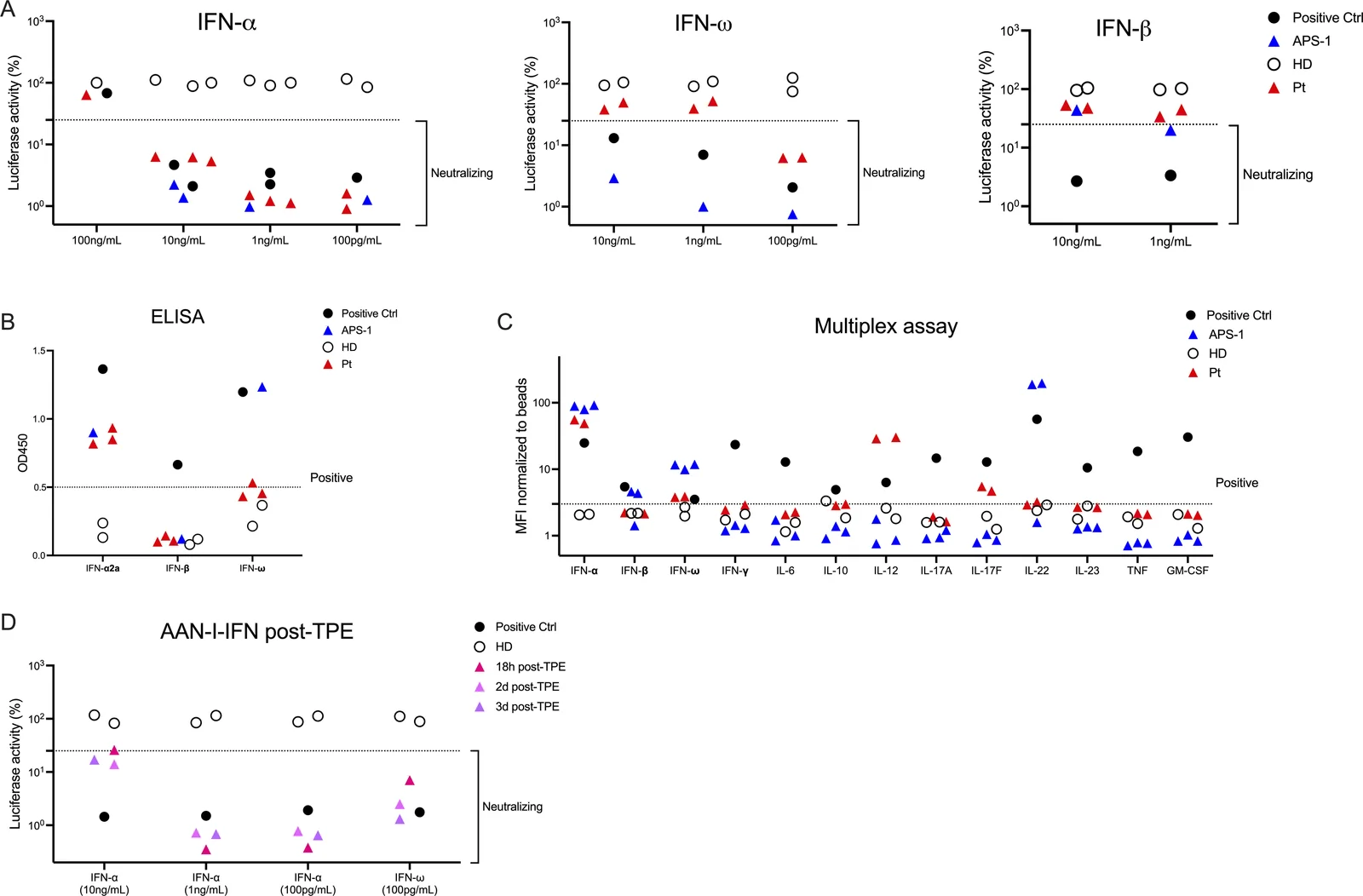
**AAN-I-IFN in the patient’s blood neutralized IFN-α and IFN-ω. (A)** A549-IFN-reporter (AIR) cells carrying the ISRE reporter were stimulated with IFN-α, -ω, or -β at the concentration indicated, with or without blood from the patient (pt), an AAN-I-IFN–positive control (positive ctrl), an APS-1 patient, or healthy donors (HD). All samples were diluted 1:20. *Renilla* luciferase activity was measured 24 h after stimulation. The results are expressed as a percentage of the mean value for HDs. Luciferase activity levels <25% that of HDs were considered to indicate neutralizing activity. Two separate blood draws from the patient were sampled. Experiments were done three times. **(B)** ELISA plates were coated with 1 μg/ml of the IFN subtypes indicated and incubated with blood samples (diluted 1:50). Anti-human IgG-HRP secondary antibodies were then added, and OD was measured at 450 nm. An OD_450_>0.5 was considered to be a positive results. Three separate blood draws from the patient were sampled. Experiment was done once. **(C)** Multiplex assay beads were incubated with blood samples (diluted 1:1,000), and the MFI was normalized against a beads-only control. Normalized MFI values > 3 were considered positive. Two separate blood draws from the patient were sampled. Experiment was done once. **(D)** AAN-I-IFN neutralization tested with patient’s blood collected at the indicated time points as described in A. Three separate blood draws from the patient were sampled. Experiment was done once.

**Figure S1.**
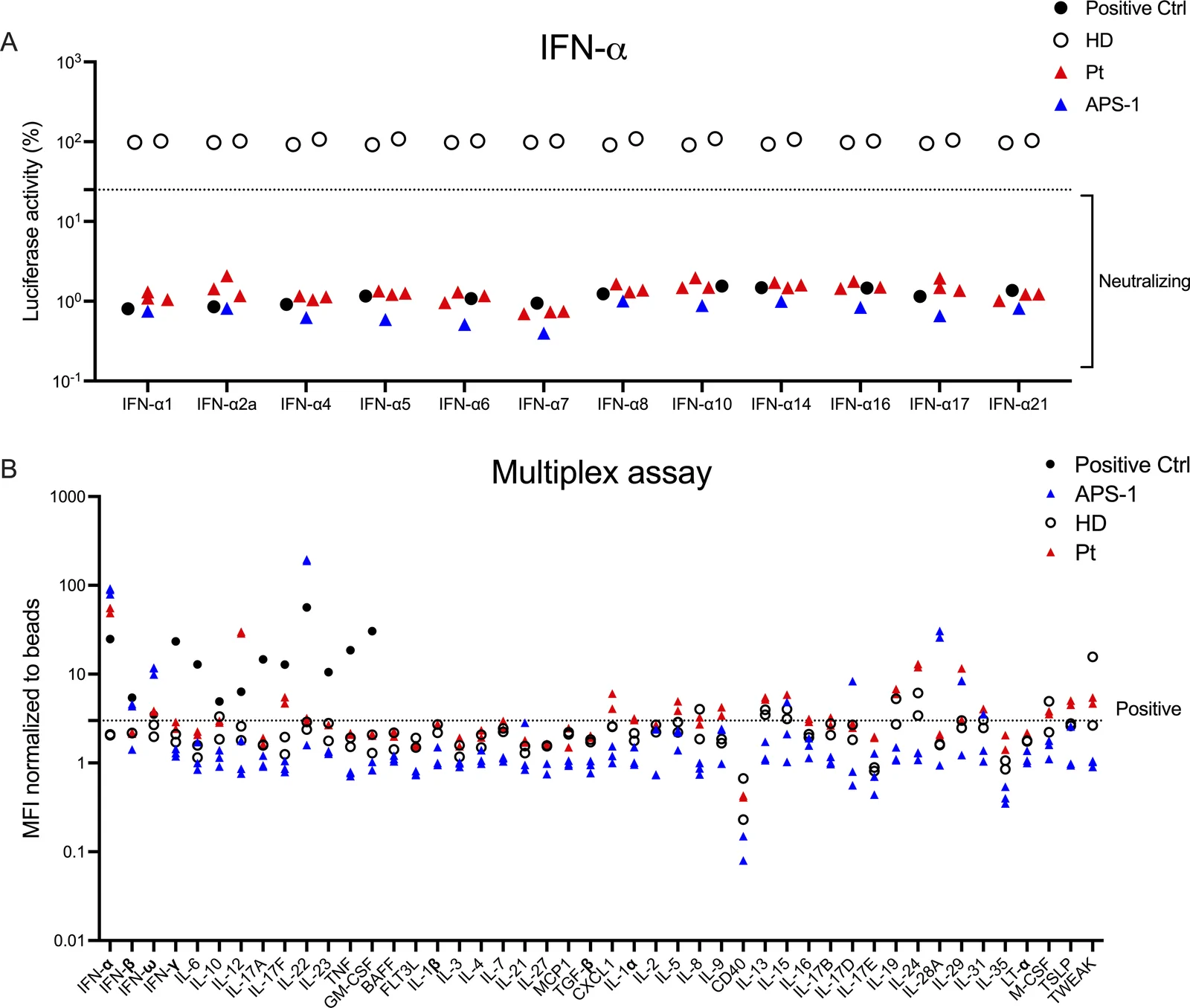
**Characterization of AAN-I-IFN in the patient. (A)** AIR cells were stimulated with the 12 subtypes of IFN-α at a concentration of 1 ng/ml, with or without blood from the patient (pt), an AAN-I-IFN–positive control (positive ctrl), an APS-1 patient, or healthy donors (HD). All samples were diluted 1:20. *Renilla* luciferase activity was measured 24 h after stimulation and expressed as a percentage of the mean value for HDs. Luciferase activity levels <25% of HD values were considered to indicate neutralizing activity. Three separate blood draws from the patient were sampled. Experiment was done once. **(B)** Multiplex assay beads were incubated with blood samples, and MFI values were normalized against the beads-only control. Normalized MFI values > 3 were considered positive. Two separate blood draws from the patient were sampled. Experiment was done once. AIR, A549-IFN-reporter.

**Figure 2.**
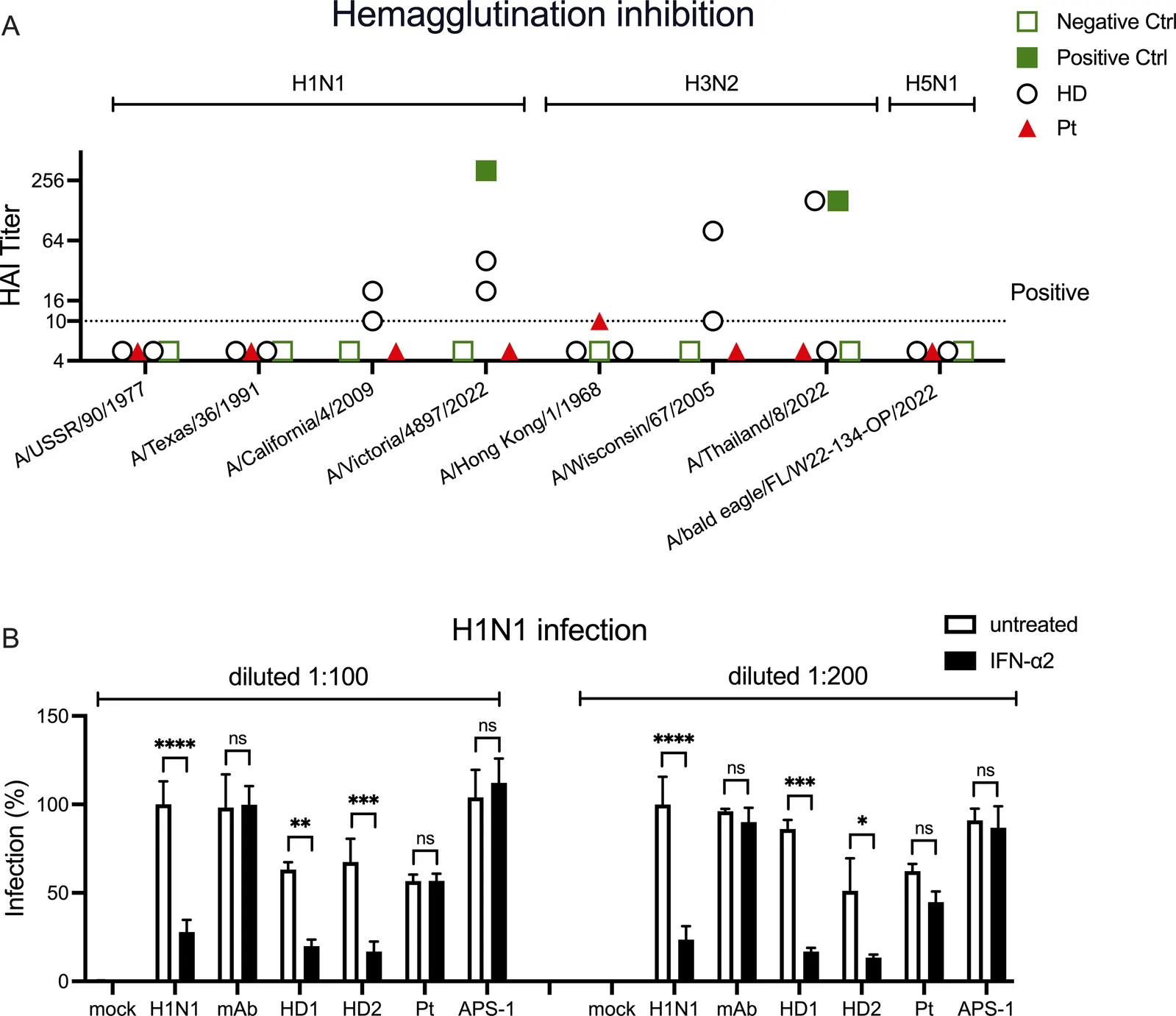
**The patient’s blood is seropositive for H3N2 and blocked IFN-α function *in vivo*. (A)** We tested for HAI assay by treating patient (Pt) and healthy donor (HD) blood with receptor destruction enzyme (RDE) and diluting 1:10 before mixing with the H1N1, H3N2, and H5N1 IAVs at the indicated titers, together with 0.5% Turkey red blood cells. Blood from naive mice and mice immunized with H1N1 (A/Victoria/4897/2022) or H3N2 (A/Thailand/8/2022) was used as negative and positive controls. Experiment was done once. **(B)** A549 cells were incubated overnight with 50 pg/ml exogenous IFN-α2 with or without anti–IFN-α2 monoclonal antibody (mAb), patient blood (Pt), healthy donor blood (HD1 and HD2), or APS-1 patient blood (APS-1) at indicated dilution, and then infected with influenza A/California/04/2009 virus expressing NS1-mCherry (CalNSmCherry) at an MOI of 1. The percentage of the cells infected was determined 24 h after infection with a Celigo (Nexcelcom) imaging cytometer. The percentage of infection was normalized against cells infected without IFN-α2 treatment. The dotted line at 26.5% indicates the mean percentage of cells infected after treatment with IFN-α2 only. Experiments were done twice and paired *t* test was performed (P values: ****<0.0001; ***<0.001; **<0.01; *<0.05; ns > 0.05).

We first tested the blood sample collected from the patient on day 3, before TPE/CRRT, for AAN-I-IFN neutralizing IFN-α, -β, and -ω in a luciferase reporter assay. The patient’s blood (diluted 1:20) neutralized 10 ng/ml IFN-α and 100 pg/ml IFN-ω, but not IFN-β (Fig. 1 A). It also neutralized all 12 subtypes of IFN-α tested (Fig. S1 A). ELISA and multiplex assays revealed high levels of IgG-binding IFN-α, intermediate levels of IgG binding IFN-ω, and no detectable IgG-binding IFN-β, consistent with neutralization assay results (Fig. 1, B and C). High IgG levels binding IL-17F, IL-22, possibly CXCL1, IL-5, IL-9, TSLP, and TWEAK were detected, but no IgG binding the other 36 cytokines tested (Fig. S1 B). We then tested the patient’s blood samples 18 h, 2 days, and 3 days after TPE treatment. Anti–IFN-α titer decreased but was still detectable 18 h after TPE. (Fig. 1 D). Serological tests were performed for IAVs. The patient’s blood samples were positive for A/Hong Kong/1/1968 by hemagglutination inhibition (HAI) assay, indicating exposure to seasonal H3N2 (Fig. 2 A). Finally, in the presence of blood from the patient, IFN-α failed to inhibit IAV replication in A549 cells, indicating that the AAN-I-IFN blocked the antiviral function of IFN-α (Fig. 2 B).

We report a fatal case of H5N1 pneumonia in a 71-year-old man with blood AAN-I-IFN neutralizing 10 ng/ml IFN-α and 100 pg/ml IFN-ω. In the general population, autoantibodies neutralizing these concentrations of IFN-α and IFN-ω are found in only 0.5% of individuals aged between 65 and 75 years and 1% of individuals aged >70 years (Bastard et al., 2021a). We previously showed that individuals with AAN-I-IFN have a very high risk of critical seasonal IAV pneumonia (OR: 10–100) (Zhang et al., 2022b). Causality between AAN-I-IFN and fatal H5N1 infection in this patient is therefore plausible. The patient was seropositive for seasonal H3N2, indicating that he had controlled seasonal IAV infections without vaccination. His AAN-I-IFN probably emerged when he was already seropositive for seasonal H3N2 (Fernbach et al., 2024; Bastard et al., 2023). The patient had no clinical history of severe infection or autoimmune disease, like many patients with AAN-I-IFN in this age group (Bastard et al., 2024). Similarly, like most individuals with AAN-I-IFN, he had autoantibodies neutralizing IFN-α and/or -ω, but not -β, and might therefore have benefited from IFN-β therapy, if administered early in infection and in combination with antiviral therapies.

We and others have shown that AAN-I-IFN underlie a growing range of severe infections of emerging and circulating viruses (Bastard et al., 2024; Hale, 2023; Busnadiego et al., 2022). This fatal case of H5N1 infection has broad clinical and biological implications. It suggests that type I IFNs may contribute to innate and intrinsic immunity to emerging viruses, including zoonotic viruses, such as avian IAV. AAN-I-IFNs are common in the general population and therefore constitute a substantial threat to public health (Bastard et al., 2021a). We also recently identified a dominant-negative mutation in *IFNAR1* (p.Pro335del), encoding the type I IFN receptor, which is remarkably common in South China (0.6–2%). This mutation impairs the response to IFN-α and -ω, but not -β, as in patients with AAN-I-IFN. Heterozygous carriers of this mutation are vulnerable to infections with various viruses, including SARS-CoV-2 (Al Qureshah et al., 2025). They may also be vulnerable to H5N1, H7N9, or other avian IAVs. New cases of H5N1 infections are increasing in the recent years (Siegers et al., 2025). Since October 2024, four other hospitalized cases of H5N1 infection have been documented in North America (Jassem et al., 2025; CDC, 2025), some of which might perhaps have been caused by inborn errors of type I IFN immunity or AAN-I-IFN (Langlois and Casanova, 2025).

The initial replication of avian IAV in humans is suboptimal due to the lack of mammal-adaptive mutations of the viral genes. However, crippled type I IFN immunity, due to inborn errors or AAN-I-IFN, may boost replication sufficiently for an avian IAV to generate mutations facilitating adaptation to mammals (Langlois and Casanova, 2025; Spieler et al., 2020). Sequencing of the influenza A/Louisiana/12/2024 H5N1 virus responsible for this case revealed low-frequency mutations in the HA segment (mixed amino acid populations at positions A134V, N182K, and E186D) associated with an increase in binding to human-type cell receptors (CDC, 2024). Previous reports of *MX1* variants in patients with severe H7N9 infections suggest that variants of a single ISG may facilitate cross-species transmission (Chen et al., 2021; Hale et al., 2010; Casanova and Abel, 2024). However, the very rare human MX1 variants are unlikely to facilitate human-to-human transmission, as the virus remains MX1 sensitive and cannot infect the predominantly MX1 wildtype population. By contrast, the common presence of AAN-I-IFN and IFNAR1 p.Pro335del may enable the virus to undergo selection for more efficient human-to-human transmission if propagated in a cluster of affected individuals, paving the way for zoonotic virus pandemics.

## Materials and methods

Written informed consent was obtained in LSU Health Shreveport in accordance with the approval of the institute review board (protocol number 2899). AAN-I-IFN neutralization (Groen et al., 2024, 2025), ELISA (Gervais et al., 2024a), multiplex assays (Bastard et al., 2020), IAV infection assay (Zhang et al., 2022b), and HAI assays (Aydillo et al., 2021), were performed as previously described.

### Online supplemental material

Fig. S1 provides more information on the autoantibodies neutralizing different type I IFN subtypes (A) and cytokines (B).
